# No Impact of Calorie or Unit Information on *Ad Libitum* Alcohol Consumption

**DOI:** 10.1093/alcalc/agx066

**Published:** 2017-09-18

**Authors:** Olivia M Maynard, Tess Langfield, Angela S Attwood, Emily Allen, Imogen Drew, Alex Votier, Marcus R Munafò

**Affiliations:** 1 UK Centre for Tobacco and Alcohol Studies, School of Experimental Psychology, University of Bristol, 12a Priory Road, Bristol BS8 1TU, UK; 2 MRC Integrative Epidemiology Unit at the University of Bristol, 12a Priory Road, Bristol BS8 1TU, UK; 3 Department of Public Health and Primary Care, University of Cambridge, Strangeways Research Laboratory, Worts Causeway, Cambridge CB1 8RN, UK

## Abstract

**Aims:**

To investigate the impact of unit and calorie information on drinking behaviour in an *ad libitum* taste test paradigm.

**Methods:**

In this experimental human laboratory study, participants were randomized to one of four conditions, balanced by gender, using a 2 (unit information: present vs. absent) × 2 (calorie information: present vs. absent) between-subjects design. The percentage of beer consumed during the taste test was the primary outcome measure.

**Results:**

Among this largely undergraduate student population, we found no evidence that either unit or calorie information impacted alcohol consumption in an *ad libitum* taste test. A manipulation check indicated that few of the participants receiving either unit and/or calorie information could accurately recall the number of units and/or calories in the beverages provided to them, indicating low levels of engagement with this information. Analysis of qualitative reactions to calorie and unit labelling indicated possible negative unintended consequences of calorie and unit information, including using unit information to facilitate consumption of higher strength beverages, and calorie information to reduce food consumption prior to a drinking episode.

**Conclusion:**

We find no evidence to support an effect of unit or calorie information, a public-health initiative supported by the alcohol industry, on drinking behaviour. It is possible that compulsory unit and calorie labelling, at least in the numeric format used here, would have no effect on alcohol intake and may even have some negative unintended consequences among certain populations.

## INTRODUCTION

In 2016, 30% of adults in the UK reported consuming more than 4 units of alcohol if male and 3 units if female on their heaviest drinking day in the past week (Office for National Statistics, 2017). This may be particularly problematic among young people; a recent review suggested over 20% of university students in the UK and Ireland exceed weekly sensible drinking limits (Davoren *et al.*, 2016). Research indicates that young social drinkers have a poor understanding of the unit content of their drinks (De Visser and Birch, 2012). As part of the 2011 Public Health Responsibility Deal, the UK alcohol industry voluntarily committed to labelling 80% of alcoholic products with unit information by December 2013, although a recent review found that this target had not been met (Institute of Alcohol Studies, 2015). Providing unambiguous unit information on alcohol products may serve to increase knowledge about alcohol consumption and ultimately help to reduce alcohol consumption. However, it is also possible that this information may have negative unintended consequences, with evidence that young social drinkers will use this information to choose the strongest drinks for the lowest cost (Jones and Gregory, 2009).

The calorie content of alcoholic beverages is also not well understood by consumers. For adults who drink, alcohol is estimated to account for approximately 10% of calorie intake (Shelton and Knott, 2014) while survey data suggests that between 60% and 80% of adults either underreport or are unaware of the number of calories in a glass of beer and wine respectively (Royal Society for Public Health, 2014). Calorie labelling is ubiquitous on soft drinks and food products, but typically absent on alcoholic beverages. Research on calorie labelling on food and non-alcoholic beverages suggests that these labels reduce food consumption (Gelobter *et al.*, 2010; Roberto *et al.*, 2010, 2012; Bollinger *et al.*, 2011) with calorie labels having greater behavioural influence among females (Heiman and Lowengart, 2014) and those with dietary goals (Miller and Cassady, 2012). Given evidence in support of calorie labelling for food and non-alcoholic beverages being an effective strategy for encouraging healthier diets (Campos, Doxey & Hammond, 2011), providing calorie information on alcohol products may also be an effective method of reducing alcohol consumption. This may occur by encouraging drinkers to switch from higher calorie/higher strength beverages to lower calorie/lower strength beverages, or encouraging drinkers to consume less overall. In the long term, requiring calorie labelling on alcoholic beverages may also encourage the alcohol industry to produce lower calorie beverages, leading to the production of lower strength beverages, as alcohol strength is highly correlated with calorie content. There is evidence of public support for calorie information on alcoholic beverages (Nikolaou *et al.*, 2015; Annunziata *et al.*, 2016). However, calorie information may also have negative unintended consequences. For example, those who are motivated to drink but also limit their calorie intake may reduce food consumption prior to drinking. This motivation to consume is relatively unique to alcoholic beverages and it therefore cannot be assumed that results from studies on food and non-alcoholic beverages labels will translate to alcoholic beverages.

In 2015, the UK government All Party Parliamentary Group on Alcohol Misuse recommended that ‘every alcohol label should include an evidence-based health warning as well as describing the product's nutritional, calorific and alcohol content (page 7)’. Given the potential unintended consequences of unit and calorie labelling for alcoholic beverages, it is important to assess the impact of this proposed legislative change on drinking behaviour. Here, using an *ad libitum* taste test paradigm, we investigated whether unit and/or calorie information impacts drinking behaviour in the laboratory. The *ad libitum* taste test procedure has good construct validity, with alcohol intake during the taste test correlated with self-reported typical alcohol consumption (Robinson *et al.*, 2017).

## METHODS

### Study design

In this experimental human laboratory study, participants were randomized to one of four conditions, balanced by gender, using a 2 (unit information: present vs. absent) × 2 (calorie information: present vs. absent) between-subjects design. The volume of beer consumed during an *ad libitum* taste test was the primary outcome measure. The study protocol including data analysis plan was pre-registered on the Open Science Framework (https://osf.io/58rw7/).

### Participants

We recruited social alcohol consumers (defined as drinking at least two units per week and no more than 35 units per week if female or 50 units per week if male). Participants were recruited from a database at the University of Bristol, which included students, staff, and the public. Participants were required to be at least 18 years of age, in good psychiatric and physical health, and to like beer. Exclusion criteria included use of illicit drugs (except cannabis), family history of alcoholism and recent consumption of alcohol (within 24 h of the test session). Participants were reimbursed £5. The study was approved by the Faculty of Science Research Ethics Committee at the University of Bristol (ethics approval code: 24,091,526,481).

### Measures and Materials

#### *Ad libitum* taste test

Participants were given two 284 ml (half pint) glasses of beer (Stella Artois, 43 calories/100 ml, 4.8% ABV). Both beers were served chilled and in identical glasses. The glasses were presented on an A4 laminated sheet, with ‘Beer 1’ and ‘Beer 2’ clearly demarcated. Participants were informed that they were to taste and rate these beers on a 10 point Likert-type scale from 1 (Not at all) to 10 (Extremely) for 10 descriptors: fruity, smooth, sweet, refreshing, bitter, strong-tasting, gassy, pleasant, light and tasty. Except for the ‘pleasant’ and ‘tasty’ ratings, which we analysed for ‘drink enjoyment’, the taste ratings were not analysed.

#### Experimental manipulation

The experimental manipulation (i.e. calorie/unit information vs. no information) was provided to participants alongside other information about the beers (i.e. popularity of the beers), ostensibly to assist in making the taste ratings and to give the impression that the beers were different, making the ‘taste-test’ a more viable cover story. This information was presented inside an envelope to ensure that the experimenter was blind to the condition allocated to the participant. For those in the unit and calorie information condition, this information read: ‘Beer 1: 284 ml, chilled to 4°C, 128 calories, 1.4.units (4.8% ABV), most popular beer in the UK; Beer 2: 284 ml, chilled to 4°C, 128 calories, 1.4.units (4.8% ABV), 5th most popular beer in the UK’. Those in the no calorie and/or no unit information condition received the same information but with the relevant information removed.

#### Questionnaire measures

After the taste test, participants were asked to imagine a hypothetical scenario in which they could only consume each beer for an evening, and were required to indicate how many half pints they would choose to consume. Alcohol craving was measured using the Alcohol Urges Questionnaire (AUQ) (Bohn *et al.*, 1995). The Positive and Negative Affect Schedule (PANAS) (Watson *et al.*, 1988) was administered to assess mood. To measure individual differences in hazardous drinking behaviour, to be adjusted for in the main analysis, we administered the Alcohol Use Disorders Identification Test (AUDIT) (Saunders *et al.*, 1993). To measure individual differences in eating behaviour, to adjust for in the main analysis, the Three-Factor Eating Questionnaire (TFEQ) was administered (Stunkard and Messick, 1985).

#### Funnelled debrief

To awareness of the aims of the taste test and study, a funnelled debrief was administered. Participants were asked to identify the ‘purpose of the taste test’ and were given seven possible options. Those who gave the answer ‘To measure how much I would consume of each drink’ were reported to be aware of the purpose of the taste test (Field and Eastwood, 2005). Participants were also asked to ‘describe the purpose of the study’ again with seven options. Those who answered, ‘To find out whether calorie and unit information influences drinking behaviour’ were reported to be aware of the purpose of the study.

#### Impact of calorie and unit labelling

To assess subjective responses to calorie and unit labelling, including unintended consequences of this information, participants were asked to report how calorie and unit information would influence their alcohol consumption.

### Procedure

Participants were invited to attend a single weekday afternoon test session, lasting 30–45 min. Upon arrival, they provided informed consent. We confirmed abstinence from alcohol using a breath test (AlcoDigital Breathalyser 3000), where a reading of 0 μg/l was required. Participants then provided basic demographic information (age, gender, education level). Outside of participant view, a 568 ml can of Stella Artois was divided between two glasses, which were then weighed to ensure equal content.

At the start of the taste test, the experimenter placed the beers in front of the participant on the laminated sheet, and handed them a sealed envelope, which they were told contained information about the two beverages. These envelopes were labelled A, B, C or D, corresponding to condition. Randomization was determined using a pre-assigned code using random number generation in MATLAB (Mathworks, version 2015a). Participants were informed they would have 10 min to complete the taste test on the computer, and to drink as much or as little as they would like to make their ratings.

After 10 min, the experimenter returned to remove the beer glasses and envelope. Participants were then asked to complete various questionnaires (AUQ, PANAS, AUDIT and TFEQ), during which time the experimenter left the room, and measured the beer remaining. Participants then completed the funnelled debrief questionnaire and a manipulation check required participants to report, or guess, the number of units and calories in each beer. Participants then completed the questions on how unit and calorie information would influence their behaviour. Finally, participants could make further comments about the impact of unit and calorie labelling and other comments about the study.

At the end of the session, participants were debriefed, reimbursed, and asked to sign a safety form. All participants were given the option of a taxi home, or to stay behind, to allow the effects of the alcohol to wear off before leaving.

### Statistical analysis

Based on a power calculation conducted in G*Power 3.1, (*d* = 0.35, 80% power, alpha = 5%), we recruited 264 social alcohol consumers. Percentage of beer consumed (calculated by weighing the beer remaining in the glass) and taste ratings were summed and averaged respectively for the two identical beers. The primary analysis was a 2 × 2 ANOVA to examine the difference in percentage of beer consumed between: (1) those participants in the calorie information and no calorie information conditions, and (2) those in the unit and no unit conditions. Analyses were conducted both with and without adjustment for age, sex, and scores on the AUDIT and TFEQ. The secondary analysis comprised four ANOVAs to examine the differences in subjective ratings of: (1) mood (AUQ), (2) alcohol craving (PANAS), (3) subjective measures of drink enjoyment and (4) intentions to consume the beverage in the future, between conditions. Sensitivity analyses excluded participants in the calorie and unit information condition whose response to the first manipulation check question regarding total calorie and unit content was more than 15% outside of the true value, participants who guessed the true purpose of the taste test, and participants who determined that the purpose of the study was to investigate the effect of calorie and unit information on drinking behaviour.

## RESULTS

### Participant characteristics

Characteristics of participants (*n* = 264) in the four conditions are shown in Table 1. There was strong evidence for a difference in the amount of beer consumed between male and female participants (*F*_(1,261)_ = 44.98, *P* < 0.001, η_*p*_^2^ = 0.26). Female participants consumed 39% (SD = 20%) of the beer, as compared with male participants who consumed 62% (SD = 27%). Only one female (0.8%) consumed more than 99% of the beer available, while 23 (17%) males did. Females had a mean AUDIT score of 10 (SD = 4), while males had a mean AUDIT score of 12 (SD = 6).

**Table 1.** Characteristics of participants in the four conditions

	No units	No units	Units	Units
	No calories	Calories	No Calories	Calories
	(*n* = 67)	(*n* = 65)	(*n* = 66)	(*n* = 66)
Female (%)	49.3	49.2	49.6^a^	50.0
Age (mean, SD)	23.0 (6.6)	22.8 (7.1)	21.4 (2.6)	22.0 (3.9)
Completed high school (%)	70.1	73.8	72.0	72.7
AUDIT (mean, SD)	10.2 (4.8)	10.8 (4.7)	11.5 (5.0)	10.6 (5.3)

^a^One participant in this condition did not disclose their gender.

Alcohol Use Disorders Identification Test (AUDIT) score indicates level of hazardous drinking, and has a possible score range from 0 to 40, with higher scores indicating greater levels of hazardous drinking. Scores of 8 or above indicate hazardous or harmful drinking.

### Primary outcome: Impact on drinking behaviour

**Fig. 1.**
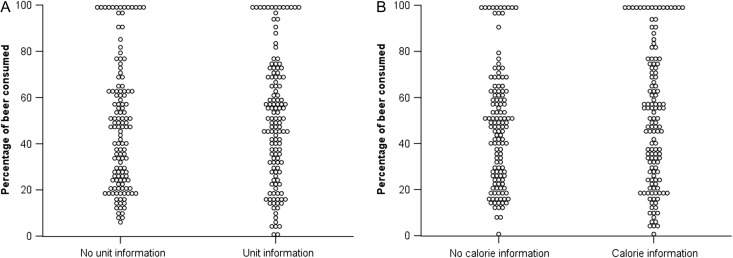
Scatter plot displaying percentage of beer consumed for participants in the (**A**) no unit information/unit information conditions and (**B**) no calorie information/calorie information conditions.

A 2(calorie information: present, absent) × 2(unit information: present, absent) ANOVA indicated that there was no clear evidence for a difference in percentage of beer consumed for participants receiving calorie information (*M* = 50% consumed, SD = 29%) as compared with no calorie information (*M* = 47%, SD = 25%; *F*_(1,260)_ = 0.88, *P* = 0.35, η_*p*_^2^ < 0.01). Given this null effect, Bayesian analyses were conducted, providing strong evidence for this finding (BF_10_ = 0.07). There was also no evidence for a difference in the percentage of beer consumed for those receiving unit information (*M* = 50%, SD = 27%) as compared with no unit information (*M* = 47%, SD = 27%; *F*_(1,260)_ = 0.646, *P* = 0.42, η_*p*_^2^ < 0.01). Again, Bayesian analyses strongly corroborated this (BF_10_ = 0.08). ANCOVA with adjustment for age, gender and scores on the AUDIT and TFEQ did not meaningfully change these results. These results are shown in Fig. 1.

### Secondary outcomes: Impact on mood, craving, drink enjoyment and drinking intentions

Multiple 2 × 2 ANOVAs on each of the secondary outcomes found no clear evidence for an impact of either unit or calorie information on positive mood, alcohol craving, drink enjoyment or intentions to consume the beverages in the future (see Table 2). There was weak statistical evidence that those participants receiving calorie information reported a more negative mood as compared with those in the no calorie information condition.

**Table 2.** Impact of unit and calorie information on the secondary outcome measures

	No unit information	Unit information	*P* value	No calorie information	Calorie information	*P* value
	*n* = 131	*n* = 133		*n* = 132	*n* = 132	
Positive mood (PANAS)	26.18 (6.62)	25.72 (7.36)	0.60	26.28 (7.11)	25.62 (6.89)	0.45
Negative mood (PANAS)	12.50 (3.27)	12.59 (3.42)	0.81	13.01 (3.85)	12.09 (2.68)	0.025
Alcohol craving (AUQ)	20.47 (8.48)	21.30 (9.35)	0.45	21.24 (8.62)	20.53 (9.24)	0.51
Drink enjoyment	6.57 (1.33)	6.30 (1.35)	0.10	6.40 (1.25)	6.48 (1.43)	0.61
Intentions to consume the beverage in the future	4.50 (2.57)	4.45 (2.43)	0.87	4.43 (2.58)	4.61 (2.41)	0.39

Values represent means (SD). The Positive and Negative Affective Schedule (PANAS) has a possible score range from 10 to 50 for each of the positive and negative components, with higher scores indicating higher levels of both positive and negative mood. The Alcohol Urges Questionnaire (AUQ) has a possible score range from 8 to 56 with higher scores indicating greater urges to consume alcohol.

### Exploratory analyses

**Fig. 2.**
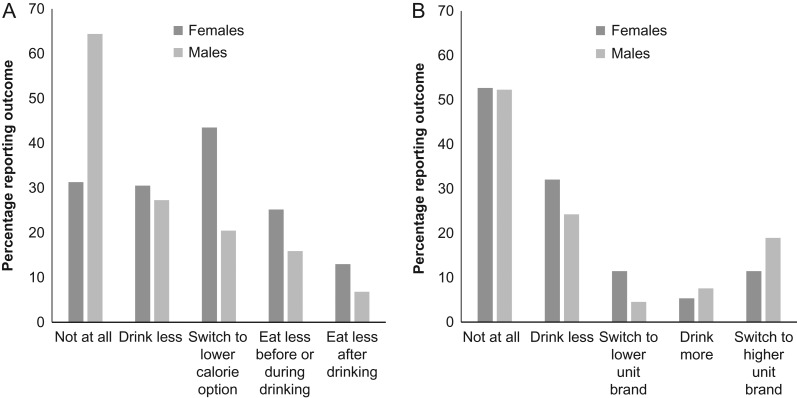
Responses to the question (**A**) ‘How would calorie information influence your alcohol consumption?’ and (**B**) ‘How would unit information influence your alcohol consumption?’.

Figure 2 shows the percentage of participants reporting the behaviours they might engage in in response to unit and calorie labelling.

We examined the relationship between dietary restraint (a subscale of the TFEQ) and percentage of beer consumed for those in the calorie information condition (*r* = −0.21, *P* = 0.02) and the no information condition (*r* = −0.19, *P* = 0.03) suggesting that those participants reporting higher levels of dietary restraint consumed less beer.

### Sensitivity analyses

#### Manipulation check

The actual number of units in each of the beers was 1.4. As shown in Table 3, there was no evidence of a difference in the number of units guessed by participants in the unit information conditions as compared with the no unit information conditions (*F*_(1, 260)_ < 0.001, *P* = 0.99, η_*p*_^2^ < 0.01). Bayesian analyses provided moderate evidence for this finding (BF_10_ = 0.14).

**Table 3.** Manipulation check for unit and calorie information

	No unit information	Unit information	No calorie information	Calorie information
	*n* = 131	*n* = 133	*n* = 132	*n* = 132
Number of units/calories^a^ guessed (mean, SD)	1.65 (1.51)	1.65 (0.88)	256.70 (219.85)	170.17 (75.40)
Guessing # units/calories^a^ correctly (%)	0.0	14.3	0.0	36.4
Guessing # units/calories^a^ within 15% of true value (%)	17.6	41.4	10.6	53.0

^a^Values represent guesses for unit content in Columns 2 and 3 and calorie content in Columns 4 and 5.

The actual number of calories in each of the beers was 128. As shown in Table 3, participants in the calorie information conditions guessed that the beers had on average fewer calories than those in the no calorie information conditions (*F*_(1,260)_ = 18.29, *P* < 0.001, η_*p*_^2^ = 0.07).

As a planned sensitivity analysis, the primary analysis was repeated excluding participants who could not report the number of units/calories (as appropriate for their condition) to within 15% of the true value. Univariate ANOVA indicated that participants in the unit information condition who reported the number of units to within 15% of the actual value consumed more of the beer (*n* = 55, *M* = 55%, SD = 28%) as compared with those in the no unit information condition (*n* = 133, *M* = 47%, SD = 27%) although there was no clear statistical evidence for a difference (*F*_(1,184)_ = 2.309, *P* = 0.13, η_*p*_^2^ = 0.012). Participants in the calorie information condition who reported the number of calories to within 15% of the actual value consumed more of the beer (*n* = 70, *M* = 52%, SD = 29%) as compared with those in the no calorie information condition (*n* = 32, *M* = 47%, SD = 25%) although again, there was no statistical evidence that this difference was meaningful (*F*_(1,200)_ = 1.536, *P* = 0.22, η_*p*_^2^ < 0.01).

#### Exclusion of those participants who guessed true purpose of taste test

Only a minority (8%) of participants correctly guessed the true purpose of the taste test. Excluding these participants had no effect on the conclusions from our primary analysis on the percentage of beer consumed. Participants who guessed the true nature of the taste test (*n* = 21) consumed 56% of the beer (SD = 27%) while those who did not guess this (*n* = 243) consumed 48% (SD = 27%).

#### Exclusion of those participants who guessed the true purpose of the experiment

Almost a quarter of participants (23%) correctly guessed the true purpose of the experiment. Excluding these participants had no effect on the conclusions from our primary analysis on the percentage of beer consumed. Participants who guessed the true nature of the experiment (*n* = 60) consumed 46% of the beer (SD = 25%) while those who did not guess this (*n* = 204) consumed 49% (SD = 28%).

### Qualitative analysis

Over half (58%) of the participants wrote a response to the free-text question ‘Do you have any other comments about calorie or unit labelling?’ We used guidelines developed by Braun and Clarke (2006) to conduct an exploratory thematic analysis of these data. From reading these answers, initial codes were identified and themes were grouped together and assessed for consistency and variability. The interpretation of the themes was primarily conducted by a single author (OMM), by re-reading the answers, consulting with colleagues and reading relevant literature. These themes were then combined to form four overarching themes: (1) incompatibility of unit and calorie labelling with drinking motivations, (2) calorie information to reduce consumption, (3) negative unintended consequences of labelling and (4) lack of knowledge.

#### Incompatibility of unit and calorie labelling with drinking motivations

##### Drinking to get drunk

Both male and female participants reported that as university students, their motivation for drinking was to get drunk.

I think, especially at university, very few people take notice of unit/calorie information, because the aim behind drinking is to get drunk, and people want to do that in whatever way possible, rather than worrying about consequences (Male, 20)

I also think that age has a huge influencing factor as I feel students will worry less about number of units and calories (Female, 20)

##### Drinking to socialize

The view that unit and calorie labelling detracts from the social aspect of drinking was also highlighted by several participants.

I think that when I’m in a situation where I choose to drink, I tend to not think about calorie content etc. of the drinks, and really just concentrate on enjoying myself (Male, 23)

I try not to listen to calorie and unit information because it distracts from the social aspect of drinking (Male, 18)

#### Calorie information to reduce consumption

Although there were a mix of opinions, generally it was reported that calorie information might make alcohol consumers think more carefully about their consumption, although this was most commonly reported by female participants.

If calorie information were more visibly displayed, I may think twice about which beers I drink. However, I have no clue! (Female, 20)

There was the view that calorie information would only affect those who were worried about their weight or who already restricted their calorie intake from food.

I have flatmates who count the calories before they drink so it will matter mostly to those who are keeping track of their weight and diet (Male, 23)

#### Negative unintended consequences of labelling

##### Unit labelling facilitates increased consumption

Due to a motivation to get drunk, many participants expressed the view that neither unit nor calorie labelling would have an impact on their behaviour. Many reported that unit labelling would serve to increase alcohol consumption. The ‘cost-effectiveness’ of alcohol was expressed by many participants as important, where unit information would assist in choosing high strength drinks for a low price.

I think for the student population, when drinking is sadly almost central to our social lives, the impact of particularly unit labelling would be to promote higher consumption of cheaper, higher unit brands (Male, 22)

I have some friends who would be influenced by a low unit beer, i.e. would not drink as it is less strong and therefore less cost-effective (Male, 20)

I would always choose the drink with more units if it was the same price (Female, 18)

##### Calorie labelling encourages food restriction

Given participants’ motivations to get drunk, some female participants reported that to achieve this, they would eat less food if made aware of the calorie content of their drinks.

In calorie-conscious people, I believe the number of calories would affect the amount they eat during the day (in anticipation of the night's drinking), but not on the specific drinks they choose (Female, 19)

##### Weighing off the unit and calories in beverages

A common theme among both male and female participants was the use of unit and calorie information to choose a drink with more units, but fewer calories.

Usually try and consume more units for less calories (Male, 22)

I think that some people (myself included) take more notice of calorie labelling in comparison to unit labelling. If a drink was lower in calories but higher in units, I would be more likely to drink that than a drink high in calories but low in units of alcohol (Female, 23)

#### Lack of knowledge

##### Calories

In general, participants reported being unaware of the number of calories in their drinks. Parallels were drawn with food products where calorie labelling is ubiquitous and it was suggested that calorie information on alcoholic beverages is a consumer right.

I think the general public are unaware of quite how many calories there are in alcohol. Perhaps because it's a drink rather than food (which is very much associated with calorie-counting) people forget it is a substance very high in calories (Female, 20)

I have never thought about calorie of drinking before, but I will concern it next time—maybe drink less to reduce caloric intake (Female 18)

I think that calorie information should be clearly labelled on alcoholic drinks, it is ridiculous that guidelines are so tight for foods but alcoholic drinks are immune (Male, 20)

##### Units

The abstract nature of units was noted by a number of participants.

I think it is very difficult for people to apply unit labelling into reality (Female, 24)

I think calorie information will have a greater impact than unit information, it is more relatable (I think)—units can seem ‘abstract’ (Female, 21)

## DISCUSSION

We found no evidence that either unit or calorie information impacted alcohol consumption in an *ad libitum* taste test. Furthermore, unit and calorie information did not impact mood, alcohol craving or intentions to consume the beers in the future. There was weak evidence that calorie information, but not unit information, reduced drink enjoyment. Despite having the unit and/or calorie information in front of them for 10 min during the taste test, participants were generally not able to recall the percentage of units or calories in their beverages, as indicated by manipulation checks. There was weak evidence that those in the information conditions who could recall the number of units and/or calories in their beverages, consumed more beer than those who did not receive this information.

Qualitative analyses indicated that, among this largely undergraduate population, the main motivation for drinking was to get drunk. Where unit information was perceived as being helpful, this was to facilitate choice of higher strength drinks. This has been shown previously among this population (Jones and Gregory, 2009) and highlights the potential negative unintended consequences of unit information among young alcohol consumers. Calorie information was expected to be effective at encouraging healthier drinking among some participants, although many believed it would affect others rather than themselves. Some participants noted that calorie information may only serve to encourage reduced food consumption before a heavy drinking episode. The concept of trading off calories and units was a common theme, with participants saying they would try to drink as many units as possible, while consuming fewest calories. Given that units and calories are highly correlated, one method of reducing unit consumption might be to promote lower calorie options. The concept of units was poorly understood by participants, with many reporting that it was too abstract. Alternative methods of presenting unit information should be explored.

In sum, we found no evidence to support an effect of unit or calorie information on drinking behaviour. Our qualitative data also provide evidence of potential negative unintended consequences of this information, such as using unit information to facilitate higher alcohol consumption. There was also some indication that calorie information would be used to reduce food consumption rather than alcohol consumption. Previous research has gone further to suggest that calorie information increases consumption intentions (Bui *et al.*, 2008). It is therefore possible that compulsory unit and calorie labelling, at least in the format presented here, would have little or no effect on alcohol intake and may even have some negative unintended consequences among certain populations.

To our knowledge, this is the first study to have used the *ad libitum* taste test task to investigate the impact of unit and calorie information on drinking and there are several potential limitations of our design. First, it is possible that the ad libitum task is not suitable, as unit and calorie information may influence drink choice, rather than drink consumption, and effects of labelling may not be evident after a single exposure. Future research should use alternative designs to test these hypotheses. Second, rather than presenting unit and calorie information on the beer bottle or glass, as might occur in a real-life drinking environment, participants were provided with information on a piece of paper for 10 min while completing the taste test. Although this was to increase the likelihood that participants read the information, the manipulation check indicated that few participants in either the calorie or unit information conditions could correctly recall the number of calories or units in their beverage to within 15% of the true value. The fact that participants failed to read the information limits our ability to claim that unit and calorie information have no effect on drinking behaviour. However, arguably this better approximates how drinkers might behave in naturalistic settings where they might not notice this information or actively avoid it. Indeed, it was noted in the qualitative phase that unit and calorie information distracted from the social element of drinking and would potentially be ignored. Third, our sample mainly consisted of undergraduates and it is possible that these findings are unique to student populations and other groups whose main motivation when drinking is to get drunk. Further research should investigate the impact of unit and calorie information among other populations of alcohol consumers. Finally, our qualitative analysis provided important context to the quantitative data, although it should be noted that the analysis of the qualitative data was exploratory and conducted by a single researcher. Future research should examine the themes outlined here in more detail.

These findings question whether mandatory unit and calorie labelling, as proposed by the All Party Parliamentary Group on Alcohol Misuse, and in principle supported by the alcohol industry in the 2011 Public Health Responsibility Deal, would be an effective alcohol control strategy. Reducing alcohol consumption will likely require a range of public-health interventions, and it is crucial to provide a strong evidence base to inform which, if any, will be effective.

## AVAILABILITY OF DATA AND MATERIAL

The data that form the basis of the results are available from the Bristol Research Data Repository (http://data.bris.ac.uk/data/), doi: https://doi.org/10.5523/bris.3fkat4syqn24i2ol2mpr199egl.

## FUNDING

This work was supported by the Medical Research Council Integrative Epidemiology Unit at the University of Bristol, which is supported by the Medical Research Council and the University of Bristol (MC_UU_12013/6 and MC_UU_12013/7). The funders had no role in study design, data collection and analysis, decision to publish, or preparation of the manuscript.

## CONFLICT OF INTEREST STATEMENT

The authors declare that they have no competing interests.

## AUTHORS’ CONTRIBUTIONS

O.M.M., T.L., E.A., A.A. and M.M. contributed to the conception and design of the study and contributed to the study protocol. T.L., E.A., I.D. and A.V. managed the day-to-day running of the study and testing of participants. O.M.M. performed the data analysis and all authors helped with data interpretation. This manuscript was written by O.M.M. and T.L. with input from all co-authors. All authors read and approved the final version of the manuscript.

## References

[agx066C1] Annunziata A, Pomarici E, Vecchio R, et al (2016) Nutritional information and health warnings on wine labels: exploring consumer interest and preferences. Appetite 106:58–69. 26939529 10.1016/j.appet.2016.02.152

[agx066C2] Bohn MJ, Krahn DD, Staehler BA (1995) Development and initial validation of a measure of drinking urges in abstinent alcoholics. Alcohol Clin Exp Res 19:600–6. 7573780 10.1111/j.1530-0277.1995.tb01554.x

[agx066C3] Bollinger B, Leslie P, Sorensen A (2011) Calorie posting in chain restaurants. Am Econ J Econ Policy 3:91–128.

[agx066C4] Braun V, Clarke V (2006) Using thematic analysis in psychology. Qual Res Psychol 3:77–101.

[agx066C5] Bui MY, Burton S, Howlett E, et al (2008) What am i drinking? The effects of serving facts information on alcohol beverage containers. J Consum Aff 42:81–99.

[agx066C6] Campos S, Doxey J, Hammond D (2011) Nutrition labels on pre-packaged foods: a systematic review. Public Health Nutr 14:1496–1506. 21241532 10.1017/S1368980010003290

[agx066C7] Davoren MP, Demant J, Shiely F, et al (2016) Alcohol consumption among university students in Ireland and the United Kingdom from 2002 to 2014: a systematic review. BMC Public Health 16:173. 26895824 10.1186/s12889-016-2843-1 PMC4759952

[agx066C8] De Visser RO, Birch JD (2012) My cup runneth over: young people's lack of knowledge of low-risk drinking guidelines. Drug Alcohol Rev 31:206–12. 22050096 10.1111/j.1465-3362.2011.00371.x

[agx066C9] Field M, Eastwood B (2005) Experimental manipulation of attentional bias increases the motivation to drink alcohol. Psychopharmacology (Berl) 183:350–7. 16235080 10.1007/s00213-005-0202-5

[agx066C10] Gelobter M, Downs JS, Loewenstein G (2010) Promoting healthy choices: information versus convenience. Am Econ J Appl Econ 2:164–78.

[agx066C11] Heiman A, Lowengart O (2014) Calorie information effects on consumers’ food choices: sources of observed gender heterogeneity. J Bus Res 67:964–73.

[agx066C12] Institute of Alcohol Studies 2015. Dead on Arrival? Evaluating the Public Health Responsibility Deal for Alcohol.

[agx066C13] Jones SC, Gregory P (2009) The impact of more visible standard drink labelling on youth alcohol consumption: helping young people drink (ir)responsibly? Drug Alcohol Rev 28:230–4. 21462396 10.1111/j.1465-3362.2008.00020.x

[agx066C14] Miller LMS, Cassady DL (2012) Making healthy food choices using nutrition facts panels. The roles of knowledge, motivation, dietary modifications goals, and age. Appetite 59:129–39. 22524999 10.1016/j.appet.2012.04.009 PMC3367081

[agx066C15] Nikolaou CK, Hankey CR, Lean MEJ (2015) Calorie-labelling: does it impact on calorie purchase in catering outlets and the views of young adults[quest]. Int J Obes 39:542–5. 10.1038/ijo.2014.162 25174452

[agx066C16] Office for National Statistics 2017 Adult drinking habits. Retrieved from: https://www.ons.gov.uk/peoplepopulationandcommunity/healthandsocialcare/drugusealcoholandsmoking/datasets/adultdrinkinghabits (Release date 3 May 2017).

[agx066C17] Roberto CA, Larsen PD, Agnew H, et al (2010) Evaluating the impact of menu labeling on food choices and intake. Am J Public Health 100:312–8. 20019307 10.2105/AJPH.2009.160226 PMC2804627

[agx066C18] Roberto CA, Shivaram M, Martinez O, et al (2012) The smart choices front-of-package nutrition label. Influence on perceptions and intake of cereal. Appetite 58:651–7. 22248710 10.1016/j.appet.2012.01.003

[agx066C25] Robinson E, Haynes A, Hardman CA, Kemps E, Higgs S, Jones A (2017) The bogus taste test: Validity as a measure of laboratory food intake. Appetite 116:223–31. 28476629 10.1016/j.appet.2017.05.002 PMC5504774

[agx066C19] Royal Society for Public Health 2014 Increasing awareness of ‘invisible’ calories from alcohol. Retrieved from: https://www.rsph.org.uk/asset/3E720285-6630-496C-95A3283D3441CBA3/.

[agx066C20] Saunders JB, Aasland OG, Babor TF, et al (1993) Development of the alcohol use disorders identification test (AUDIT). WHO collaborative project on early detection of persons with harmful alcohol consumption-II. Addiction 88:791–1. 8329970 10.1111/j.1360-0443.1993.tb02093.x

[agx066C21] Shelton NJ, Knott CS (2014) Association between alcohol calorie intake and overweight and obesity in English adults. Am J Public Health 104:629–31. 24524529 10.2105/AJPH.2013.301643 PMC4025698

[agx066C22] Stunkard AJ, Messick S (1985) The three-factor eating questionnaire to measure dietary restraint, disinhibition and hunger. J Psychosom Res 29:71–83. 3981480 10.1016/0022-3999(85)90010-8

[agx066C23] Watson D, Clark LA, Tellegen A (1988) Development and validation of brief measures of positive and negative affect: the PANAS scales. J Pers Soc Psychol 54:1063. 3397865 10.1037//0022-3514.54.6.1063

